# A Ground-Nesting Galliform’s Response to Thermal Heterogeneity: Implications for Ground-Dwelling Birds

**DOI:** 10.1371/journal.pone.0143676

**Published:** 2015-11-30

**Authors:** J. Matthew Carroll, Craig A. Davis, R. Dwayne Elmore, Samuel D. Fuhlendorf

**Affiliations:** Department of Natural Resource Ecology and Management, Oklahoma State University, 008C Ag Hall, Stillwater, Oklahoma, United States of America; Phillip Island Nature Parks, AUSTRALIA

## Abstract

The habitat selection choices that individuals make in response to thermal environments influence both survival and reproduction. Importantly, the way that organisms behaviorally respond to thermal environments depends on the availability and juxtaposition of sites affording tolerable or preferred microclimates. Although, ground nesting birds are especially susceptible to heat extremes across many reproductive stages (i.e., breeding, nesting, brood rearing), the mechanistic drivers of nest site selection for these species are not well established from a thermal perspective. Our goal was to assess nest site selection relative to the configuration of the thermal landscape by quantifying thermal environments available to a ground-nesting bird species inhabiting a climatically stressful environment. Using northern bobwhite (*Colinus virginanus*) as a model species, we measured black bulb temperature (T_bb_) and vegetation parameters at 87 nests, 87 paired sites and 205 random landscape sites in Western Oklahoma during spring and summer 2013 and 2014. We found that thermal space within the study area exhibited differences in T_bb_ of up to 40°C during peak diurnal heating, resulting in a diverse thermal landscape available to ground-nesting birds. Within this thermally heterogeneous landscape, nest sites moderated T_bb_ by more than 12°C compared to random landscape sites. Furthermore, successful nests remained on average 6°C cooler than unsuccessful nests on days experiencing ambient temperatures ≥ 39°C. Models of future T_bb_ associated with 2080 climate change projections indicate that nesting bobwhites will face substantially greater T_bb_ throughout the landscape for longer durations, placing an even greater importance on thermal choices for nest sites in the future. These results highlight the capacity of landscape features to act as moderators of thermal extremes and demonstrate how thermal complexity at organism-specific scales can dictate habitat selection.

## Introduction

Thermal environments place unavoidable behavioral and physiological constraints on all living organisms [1, 2] and determine the outcome of critical life history periods [3,4]. Consequently, fine scale thermal limitations on reproductive stages can have significant population implications [5–7]. For example, extreme heat has been shown to cause adult, embryo, and chick mortality in endothermic birds [8–10], as well as reduced hatching success and alterations of sex determination in ectothermic reptiles and amphibians [11, 12]. While it is widely acknowledged that ecological processes are scale dependent [13, 14], organisms have been shown to exploit the environment at numerous scales depending on their size, movement capabilities and life stage [5,6,15]. However, daily and hourly thermal decisions are made at comparatively finer scales which can be critical for reproductive success, fitness and survival [16–18]. Additionally, studies that include temperature as predictors of biotic responses often focus on scales that are much coarser than the scale at which organisms exploit their environment [19–21]. These discrepancies can create obstacles for understanding an organism’s sensitivity to fine scale variation in thermal patterns, ultimately hindering assessments of species responses to future climate change [22, 23].

Heterogeneity is recognized as a primary mechanistic driver of broad scale ecosystem function and biological diversity, as well as fine scale patch use by organisms [24, 25]. Studies addressing the ecological effects of heterogeneity have typically focused on spatial and temporal variation in vegetation structure, leaving other basic components of heterogeneity, such as microclimate, understudied [26]. Importantly, the spatial variation of microclimate (i.e., near ground climate) creates patterns of thermal heterogeneity across landscapes that directly impact behavior, thermoregulation, and overall fitness of organisms [2, 27, 28], as well as community assemblages [29, 30]. Within these thermally heterogeneous landscapes, specific microhabitats can also buffer against thermal extremes (e.g., ambient temperature and solar radiation) thereby augmenting the completion of reproductive stages such as nesting and incubation in birds [31, 32, 33] and reptiles [34, 35]. However, the way that patterns of thermal environments influence the decisions made by individuals remains a question of high ecological importance [20]. This lack of understanding can hinder conservation efforts required for identifying and managing thermal space critical to species persistence [36], but can be overcome by assessing both spatial and temporal aspects of site selection from a thermal perspective [15].

Nest site selection is a behavioral activity that dictates the thermal environments that embryos are exposed to and ultimately convey early and critical influences on neonate fitness [37–40]. Although factors such as nest structure and incubation activity by adults are critical for successful incubation [41, 42], landscape components can also be integral for providing a template of physical environments that can promote or constrain reproductive stages [32, 38]. Because nest sites chosen by oviparous organisms are often fixed across space and time for the duration of the incubation period they remain stationary under fluctuating environmental conditions [43, 44]. Unlike reptile species that often locate nests in subterranean microhabitats that moderate temperatures [34, 45], most bird species select above ground nest sites, which further increases their potential exposure to thermal extremes and limit their choices for thermally moderated sites [46]. Additionally, nest site selection is tightly linked to predator avoidance, and tradeoffs between predation risk and thermal environments are complex and often confounded [47–49]. As a result, studies that assess both abiotic and biotic factors as selective pressures will provide a better understanding of the mechanistic basis for nest site selection and may also elucidate how these tradeoffs may be altered due to shifts in thermal space associated with future climate change [49].

Ground-nesting bird species have been shown to be especially sensitive to high heat events at both individual and population levels [50–53]. For example, levels of solar radiation have been shown to be the greatest predictor of greater prairie chicken (*Tympanuchus cupido*) nest success in the tall grass prairie of Oklahoma [33]. Additionally, northern bobwhite (*Colinus virginianus*; hereafter bobwhite) have been shown to be vulnerable to high temperatures throughout several reproductive periods (i.e., breeding, nesting, and brood rearing) [51, 54, 55]. For example, bobwhites in the Texas Panhandle of the United States commonly experienced hyperthermic conditions (≥39°C) [56] which are known to stimulate behavioral heat removal mechanisms such as refuge seeking and gular flutter (i.e., panting) [57]. Moreover, when exposed to temperatures of 46°C for a minimum of 1 hour bobwhite eggs experience 50% mortality [58]. As a ground-nesting species facing a widespread population decline [59], bobwhites also experience reduced production and undergo population declines following high heat events in the western portion of their continental distribution [57]. Because of this, bobwhites are an ideal species for assessing the impact of local thermal environments on fine scale site selection. Furthermore, individual bobwhites commonly exist on the edge of their thermal tolerance during summer heat extremes [56, 57] and therefore, are likely to be an excellent model species for assessing future climate change impacts on ground-dwelling birds.

A key component to understanding temperature constrained biotic processes involves quantifying thermal landscapes to better understand site selection decisions by organisms at multiple scales [60]. Moreover, improving conservation efforts for thermally-sensitive species will require assessments of how both abiotic and biotic factors impact ecological processes [26]; especially at a scale that reflects behavioral responses to diurnal thermal stress. For example, gaining a better understanding on how site selection choices are promoted or constrained by thermal heterogeneity will aid in disentangling the tradeoffs between microclimate and predation risk in dictating site selection [32]. Additionally, identifying the spatiotemporal positioning of thermally buffered sites at species-specific scales will also help focus conservation efforts on maintaining landscape components that modulate thermal conditions. In this study, our primary objective was to characterize thermal heterogeneity at scales relevant to ground-nesting birds. We hypothesized that in an environment prone to high heat, birds would select nests sites with structural characteristics that would confer thermal advantages by moderating temperature extremes. Therefore, we quantified thermal properties and vegetation characteristics at bobwhite nest sites, paired microsites, and random sites in order to assess the influence of proximate thermal environments on nest site selection and to determine the magnitude of potential differences in heat loads. We also assessed how the thermal conditions of nesting may be altered due to increased heat extremes as predicted by future climate change scenarios.

## Materials and Methods

### Study area

We studied the thermal ecology of nesting bobwhites at the Packsaddle Wildlife Management Area (WMA) in western Oklahoma, USA, which is near the western periphery of the North American bobwhite distribution. Packsaddle WMA is a 7,956 ha area owned and managed by the Oklahoma Department of Wildlife Conservation. The study area is dominated by sand shinnery oak (*Quercus harvardii*), but other shrub species such as sand sagebrush (*Artemisia filifolia*), aromatic sumac (*Rhus aromatica*), and sand plum (*Prunus angustifolia*) are locally common [61–63]. Common and co-dominant herbaceous species include little bluestem (*Schizachyrium scoparium*), big bluestem (*Andropogon gerardii*), sideoats grama (*Boutelou curtipendula*), blue grama (*Boutelou gracilis*), western ragweed (*Ambrosia psilostachya*), Texas croton (*Croton texensis*) and prairie sunflower (*Helianthus petiolaris*). The study area occurs primarily on sandy Brownfield and Nobscott soil types [64], and the terrain is mainly flat with 99.8% of the landscape < 24° and 62.1% of the landscape < 4.5° in slope. From 1994–2014, the region received yearly precipitation totals that averaged approximately 554.4 mm (range; 250 mm -750 mm) [65]. Intense heat events are common during summer with mean temperatures exceeding 37.8°C on average for 25 days per year [66]. Mean maximum summer temperatures exceeding the point at which heat intake can surpass heat removal in bobwhites (39°C) have also been documented [67].

### Data collection

We captured bobwhites using funnel traps during winter and spring 2013 and 2014 and fitted each bobwhite with a 6 gram necklace VHF radio-transmitter (Advanced Telemetry Systems, Isanti, Minnesota, USA). Radio-marked bobwhites were monitored using radio-telemetry to locate nest sites and monitor nesting status. Following nest confirmation, nests were monitored daily (i.e., 6–7 days per week) during the 23 day incubation period [57] until fate (i.e., successful or unsuccessful) was determined. Nests were considered successful if at least one egg hatched (n = 54) and all other nests were considered unsuccessful regardless of the cause of failure and were assigned into a category of depredated (n = 29) or abandoned (n = 4). Based on the first and last dates that nests were located, nesting season duration lasted from 8-May to 26-September in 2013 and from 28-May to 12-August in 2014.

We measured black bulb temperature (T_bb_) to investigate the thermal environments available to nesting bobwhites across the landscape relative to those at sites selected for nesting. T_bb_ provides an approximation of thermal environments that are experienced by an organism because it simultaneously incorporates ambient temperature, solar radiation, and wind effects into one interpretable metric [68]. T_bb_ was measured using steel spheres (101.6 mm-diameter; 20 gauge thickness) painted flat black (hereafter, black bulbs), equipped with an ambient temperature (T_air_) sensor suspended in the center, and situated at ground level [56, 69]. Each black bulb was connected to a HOBO U12 data logger (Onset Corporation, Bourn, Massachusetts, USA) which recorded T_bb_ at 15 minute intervals for 24 hour periods at each thermal sampling site. The use of steel spheres as black bulbs has been a common approach for investigating the thermal aspects of site selection by gallinaceous birds, such as bobwhites, [56, 70] as well as nest site selection by lesser prairie chickens *(Tympanuchus cupido)* [32]. Because black bulbs do not mimic the feather arrangement or coloration of our study species, our T_bb_ observations do not fully reflect the actual operative temperature or body temperature of bobwhites [71]. We expect that steel sphere black bulbs are subject to greater thermal heat loads than bobwhites under the same environmental conditions given that we assumed that black bulbs had greater short wave absorptivity (~1) compared to that of bobwhites (0.78) [56, 72]. Therefore, our objective was not to directly replicate the temperatures experienced by bobwhites or their eggs, but rather, was to assess nest site selection in the context of thermal landscape heterogeneity. Thus, our use of T_bb_ measurements as a standardized proxy of the environment enabled us to examine nest selection relative to the thermal patterns of the surrounding landscape, as well as to calculate the magnitude of those differences.

We measured thermal environments at 87 nests by placing a black bulb inside each nest bowl to investigate site specific T_bb_ exposure. To avoid potential systematic bias associated with sampling successful nests later in the nesting cycle than unsuccessful nests, we standardized T_bb_ measurements for nest sites by recording T_bb_ immediately following hatching for successful nests and at the projected hatch date for unsuccessful nests [32, 73]. To determine whether bobwhites selected nest sites that were cooler than those predominantly available within this heterogeneous landscape, we measured T_bb_ at 205 landscape points distributed proportional to available vegetation types using a stratified random sampling approach in ArcGIS 10.3 (Environmental Systems Research Institute, Redlands, California, USA). Sampling of T_bb_ at landscape points was distributed regularly throughout nesting season. To control for variation in T_bb_ measured at nest sites, we simultaneously measured T_bb_ at paired sites located ≤ 2 meters from each nest. At these paired sites, black bulbs were placed at a location void of overhead cover and therefore provided a fine scale environmental control relative to nest microclimates [54, 74]. While we expected that paired sites would experience higher T_bb_ than nests because of the lack of solar radiation blockage, they provided a way to obtain a relative measure of the magnitude of differences in thermal heat loads inside the nest compared to the environment outside the nest. Therefore, while comparisons between landscape points and nests provided an assessment of scale specific nest selection from a thermal perspective, paired control sites allowed us to examine potential heat loads that were possible within close proximity to nests.

To compare site specific T_bb_ measurements with simultaneously occurring macroclimate variables, we recorded ambient temperature (T_air_) and solar radiation (S_rad_) hourly at 3 onsite meteorological stations positioned 2 meters above ground level. Weather stations were distributed in an east-west orientation to match the boundaries of the study and were spaced < 7 km apart. Measurements of T_air_ and S_rad_ recorded from the meteorological station in closest proximity to each nest, paired microsite, or landscape point were used for analysis of site-specific T_bb_. Means of hourly T_bb_ across each thermal sampling array were averaged for comparisons with hourly T_air_ measurement prior to analysis. Of the 252 days of nest monitoring in 2013 and 2014, only 30 (12%) days experienced T_air_ ≥39°C which has been identified as the point at which the heat accrual exceeds heat removal in bobwhites (i.e., hyperthermic threshold) [57]. Accordingly, fewer nests were sampled on days with T_air_ ≥39°C (n = 12) than on days with T_air_ <39°C (n = 75) due the timing of extreme heat events, nest termination and sampling logistics. Nest and landscape T_bb_ were sampled under a similar range of T_air_ especially during periods of comparatively high temperatures which was a goal of our study (nest range: 7.31–43.30°C; landscape range: 19.27–43.30°C).

Vegetation structure is a primary driver associated with avian nest site selection and is a proxy for both protection from predation and thermal stress [75]. Therefore, we collected vegetation data at bobwhite nest sites and random landscape sites to examine site specific factors that could influence both thermal environments and predation exposure. Vegetation height and estimated percent cover of grass, forb, woody, litter, and bare ground cover were measured at each sampling point within a 0.5^2^ meter quadrat (modified from Daubenmire) [76] centered over each black bulb (32). To assess visual obstruction from terrestrial predators we used a Nudds board separated into 12 decimeter intervals [77] recorded at a distance of 7 meters from the nest site or landscape point [78]. We also measured the angle of obstruction directly above each sampling site (i.e., nest or landscape) given that vegetation can influence microclimate (i.e., blockage of solar radiation) and potential detection by predators [70]. Overhead angle of obstruction was quantified by aiming a digital carpenter’s level affixed on a 2 meter pole at the top of the nearest vegetation in each of the 8 cardinal and sub-cardinal directions and recording the angle reading at each sampling point [79]. Vegetation sampling was conducted following nest termination to reduce disturbing nesting bobwhites and to standardize the timing of measurements across all nest sites [80].

### Analyses

To depict thermal environments available to bobwhites during the nesting season, we modeled T_bb_ as a function of T_air_, S_rad_, and their interactions using regression analysis. Thus we accounted for T_bb_ being measured on different days. For all sites, T_bb_ was averaged by hour within diurnal periods (9:00–19:00) for each black bulb. We assessed the magnitude of differences in T_bb_ among nest sites and landscape sites by comparing standardized relative differences between T_bb_ and simultaneously recorded T_air_ (i.e., T_bb_−T_air_) experienced during the sampling period.

To evaluate the effects of *a priori* abiotic and biotic parameters on bobwhite nesting, we developed predictive models with nest fate (i.e., successful or unsuccessful) as a dependent variable based on T_bb_, visual obstruction, angle of obstruction and vegetation height as dependent variables using logistic regression and AIC model selection. Nest fates were classified as “0” for successful nests (≥1 egg hatched) and “1” for unsuccessful nests (0 eggs hatched). We assessed possible collinearity among covariates with a Pearson’s correlation test prior to analysis (range; -0.17–0.45), and each were subsequently included in candidate models. Candidate models were ranked using AIC model selection and top models with DAIC ≤ 2 were considered to have similar explanatory power [81]. We also converted model-averaged coefficients to odds ratios and 95% confidence intervals to indicate relative importance of variables and to provide an assessment of effect size. Thermal variation between successful and unsuccessful nests was analyzed using analysis of variance (ANOVA) [82] to compare T_bb_ experienced on days with maximum T_air_ < 39°C (i.e., less extreme) and T_air_ ≥ 39°C (i.e., more extreme), respectively. We used 39°C as a threshold because it is the temperature at which heat loss is outpaced by heat accrual in bobwhites, resulting in the avoidance of thermal space exceeding 39°C [57, 83], and therefore confers biological relevance for the species in this study. This threshold has also been used in studies on the thermal ecology of other gallinaceous species such as greater prairie chickens *Tympanuchus cupido* [32].

We examined variation in vegetation parameters among nest sites and landscape points using ANOVA [82]. Visual obstruction, angle of obstruction, vegetation height, and percent cover of bare ground, litter, grass, forb, and woody plants were included as site specific variables that potentially influence nest selection through moderating thermal conditions, predation avoidance, or both. Differences between groups were considered significant at the p < 0.05 level for all analyses.

We used simple linear regression of T_air_ and T_bb_ measurements from this study to project T_bb_ under future climate scenarios in order to examine potential alterations in thermal space relevant to ground nesting birds. We used these simple linear model outputs rather than merely adding predicted T_air_ increases onto observed T_bb_ in order to better characterize potential changes in site-specific nest and landscape microclimates associated with future climate change. Therefore, we used models that were based on the distribution of T_bb_ observed in this study to elevate the accuracy and real-world applicability of our predictions. Projected T_air_ used to model future T_bb_ were obtained by averaging climate models for both high and low end of century (2080) carbon dioxide emission scenarios for western Oklahoma (www.climatewizard.org) [84]. According to these average ambient temperature increases, T_air_ at the study area will increase by 2.7°C and 4.6°C for low and high emission scenarios, respectively. We compared our models of future T_bb_ at nests and landscape points to investigate whether thermal conditions differed across the same ranges of T_bb_ experienced, thus providing unbiased assessment of relative thermal conditions between groups.

### Data availability

Data are available from the Dryad Digital Repository: http://dx.doi.org/10.5061/dryad.1j6sk

### Ethics statement

Procedures for capture and handling methods used in this study were reviewed and approved by The Institutional Animal Care and Use Committee at Oklahoma State University (Protocol No.: # AG 11–22). Permission to capture, handle and monitor bobwhites on the study area was granted by the Oklahoma Department of Wildlife Conservation, which owns and manages the Packsaddle Wildlife Management Area as well as numerous other public land tracts in Oklahoma. We employed capture and monitoring techniques that have been commonly used in studies on bobwhites, including the use of walk-in funnel traps, attachment of radio-collars < 7 grams in weight, and monitoring via radio-telemetry [56, 62, 79].

## Results

**Fig 1:**
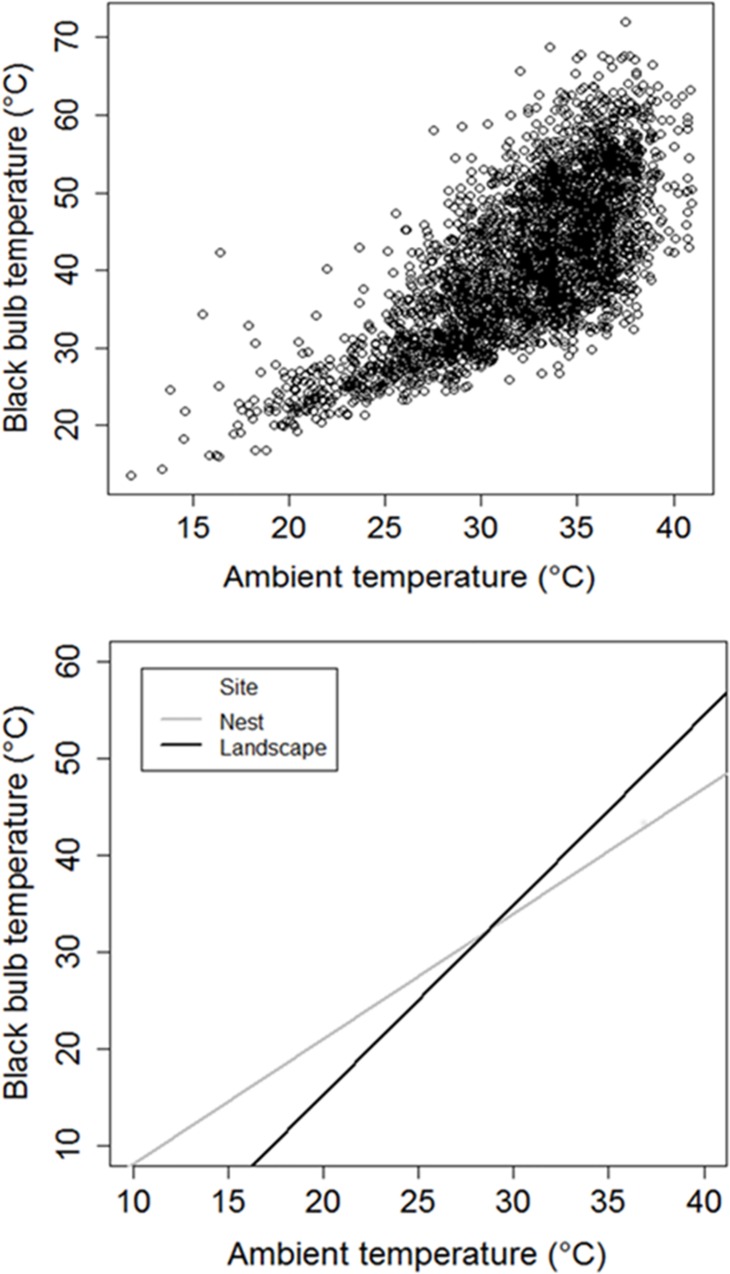
Within a thermally heterogeneous landscape, nest sites moderate thermal environments, especially during high heat. (A) Distribution of diurnal black bulb temperature (T_bb_) observed from 09:00–19:00 h (n = 3,212) and (B) linear models of T_bb_ as a function of ambient temperature (T_air_) recorded during the full sampling period (00:00–24:00 h) (B) at northern bobwhite nest and landscape sites at the Packsaddle WMA, Oklahoma, USA (2013–2014) (n = 7,008).

We found that the landscape exhibited substantial thermal heterogeneity with differences in T_bb_ ranging by up to 40°C when T_air_ > 35°C (Fig 1A). Within this thermally heterogeneous landscape, nest sites moderated T_bb_ substantially more than locations on the surrounding landscape which exhibited the potential to reach T_bb_ > 70°C (Fig 1A and 1B). Models of nest and landscape site T_bb_ showed that T_air_ and S_rad_ recorded at meteorological stations and their interaction were effective at explaining the variation in fine scale T_bb_ measurements (86%) (Table 1); however, site specific differences in microclimates were likely driven by fine scale vegetation cover.

**Table 1:** Model outputs for black bulb temperature (T_bb_) as a function of ambient temperature (T_air_) and solar radiation (S_rad_) at northern bobwhite nest sites and landscape sites at the Packsaddle WMA, Oklahoma, USA (2013–2014) (n = 7,008).

Site Modeled	Intercept	Slope Parameter			Fit (R^2^)
		T_air_	S_rad_	T_air_ X S_rad_	
Nest*	2.39 (±0.43)	0.90 (±0.018)	0.00097 (±0.0012)	0.00030 (±0.000044)	0.86
Random*	-1.96 (±0.57)	1.03 (±0.021)	0.0091 (±0.0017)	0.00025 (±0.000052)	0.86

* Denotes significance at the level of p < 0.001.

**Fig 2:**
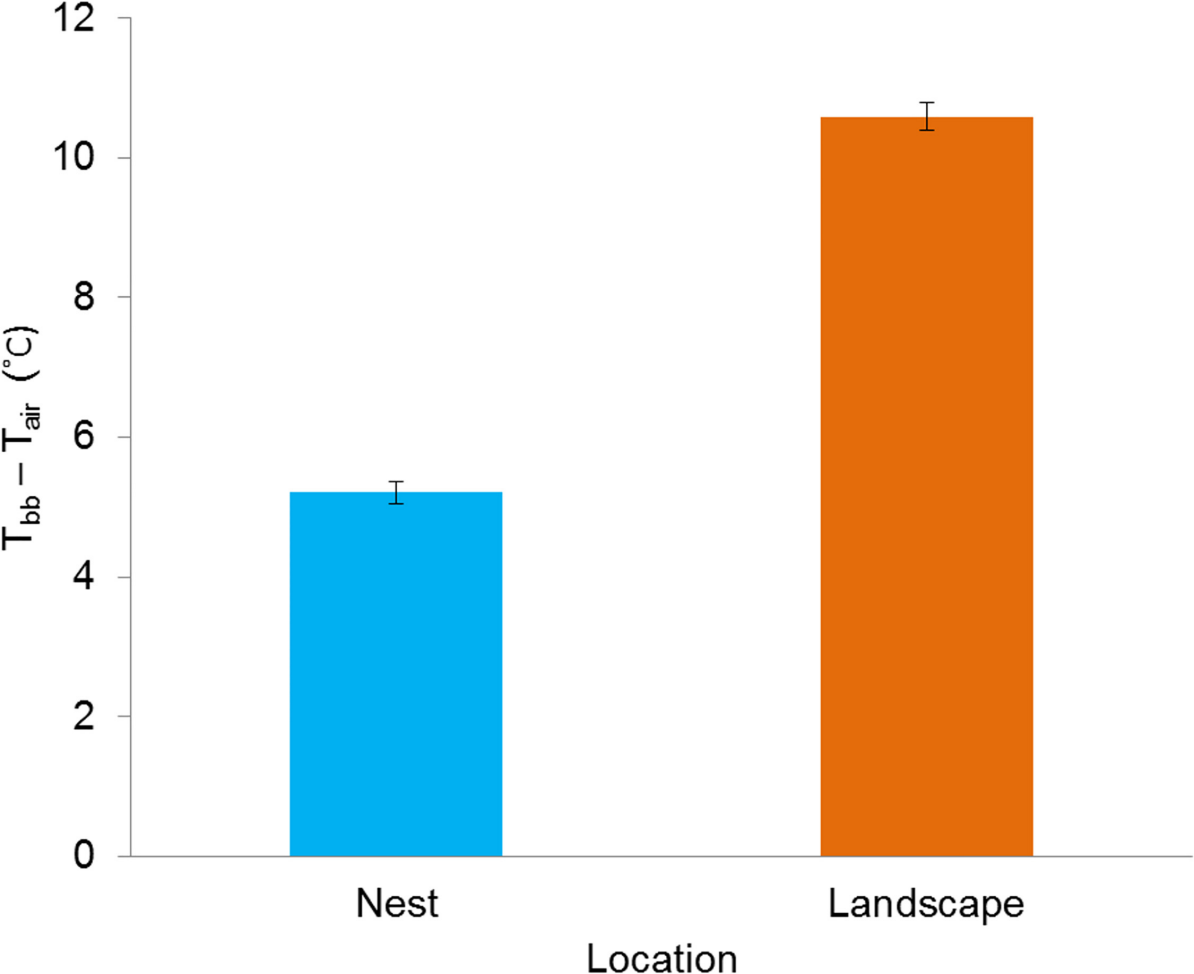
Nest sites substantially decouple thermal environments from the landscape through reduced amplification of heat loads. Differences (T_bb_−T_air_) between diurnal black bulb temperature (T_bb_) and ambient temperature (T_air_) (±SE) measured from 09:00–19:00 h at northern bobwhite nest (n = 87) and landscape sites (n = 205) at the Packsaddle WMA, Oklahoma, USA (2013–2014).

**Fig 3:**
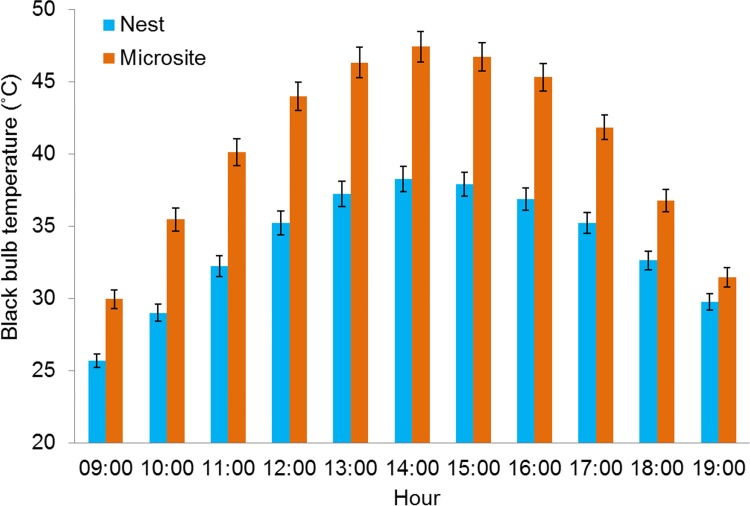
Thermal environments at proximate (within 2 m) paired control sites greatly exceed those at nest sites. Mean black bulb temperature (T_bb_) (±SE) measured from 09:00–19:00 h at northern bobwhite nests (light gray) (n = 87) and paired control sites (dark gray) (n = 87) at the Packsaddle WMA, Oklahoma, USA (2013–2014).

Nest sites acted as buffers against thermal conditions occurring on the surrounding landscape by remaining warmer at T_air_ < 28°C; yet cooler than landscape sites at T_air_ > 28°C (Fig 1B). Furthermore, we observed that the thermal buffering provided by nest sites substantially decoupled nests from surrounding conditions by reducing the amplification of T_bb_ relative to that of landscape (Fig 2). Specifically, standardized mean differences (±SE) between T_bb_ and T_air_ (i.e., T_bb—_T_air_) were more than twice as much at landscape sites (5.4°C greater) during diurnal periods (09:00–19:00) (Fig 2). Under the assumption that nest and landscape T_bb_ remained relatively consistent within 15 minute sampling periods, this difference of 5.4°C would result in an additional 1,620 degree-minutes of additional heat loads during the hottest parts of the day (11:00–16:00 h) (i.e., 5.4°C x 5 h x 60 min/h) [54]. Although nest sites moderated microclimates more than the surrounding landscape, we also observed the potential for extreme T_bb_ at sites selected for nesting. As expected, we observed temporal differences between nest T_bb_ and paired control site T_bb_ throughout the day, however, differences were substantial (Fig 3). Specifically, discrepancies in T_bb_ increased incrementally and peaked during the afternoon concomitant to daily T_air_ and S_rad_ maximums. Not only was average nest T_bb_ cooler than at paired sites or landscape sites, maximum T_bb_ recorded at nests was more than 10°C less (61.9°C) than at microsites (72.1°C) or landscape points (72.1°C).

**Fig 4:**
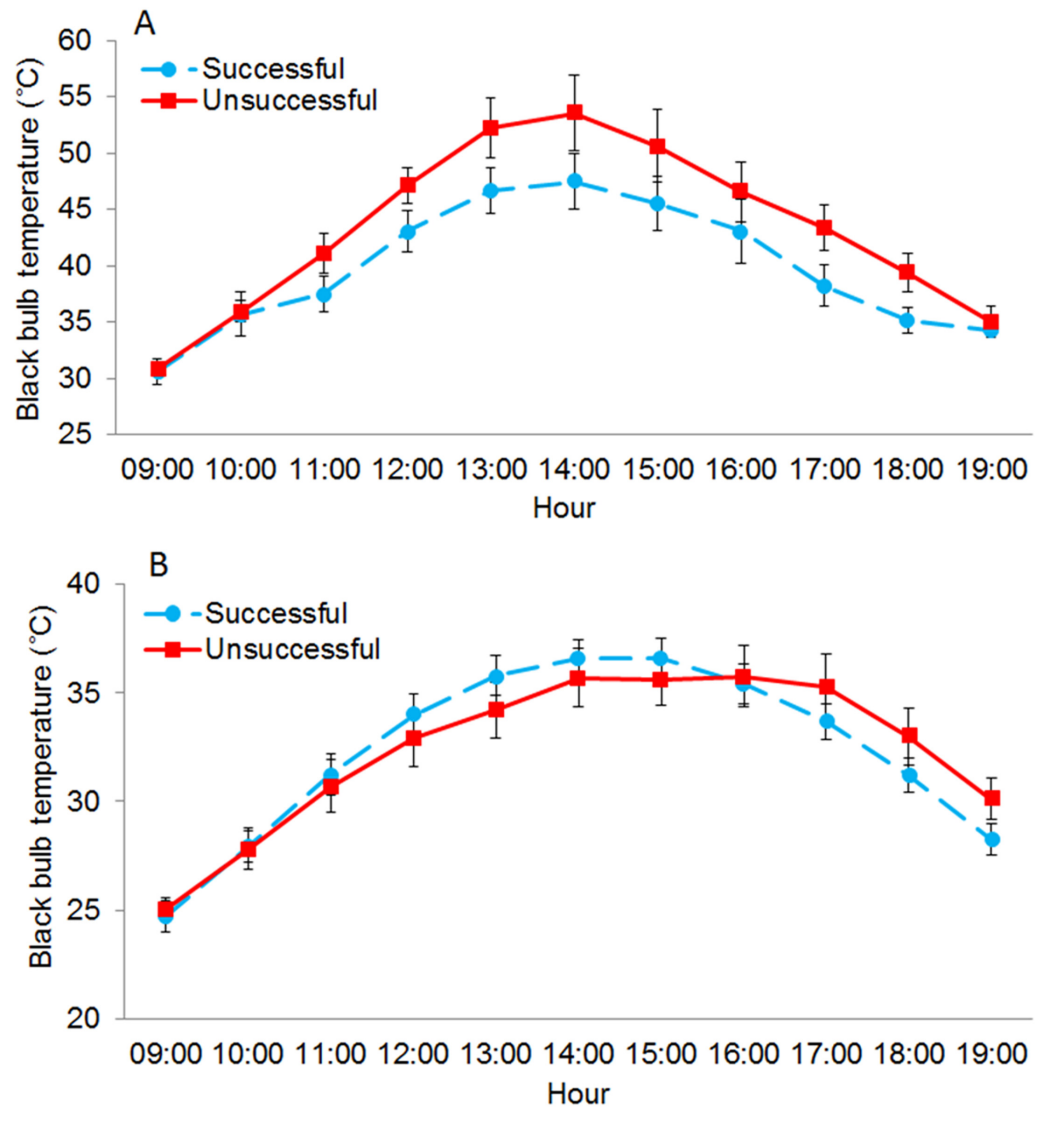
Successful nest sites moderated thermal environments more than unsuccessful nests during extreme heat. (A) Mean black bulb temperature (T_bb_) (±SE) measured from 09:00–19:00 at successful (n = 54) (dashed line) and unsuccessful (n = 33) northern bobwhite nests (solid line) on days when maximum ambient temperature (T_air_) was ≥ 39°C (n = 12) and (B) < 39°C (n = 75) at the Packsaddle WMA, Oklahoma, USA (2013–2014).

Logistic regression models identified T_bb_ as the primary single variable predicting nest success (p < 0.05) and the odds of nests being unsuccessful increased with increases in T_bb_ (i.e., hotter nest sites). Specifically, the candidate model containing nest as a lone variable produced an AIC ranking that was substantially better (DAIC ≥16) than any other models containing single vegetation variables (i.e., visual obstruction, angle of obstruction or vegetation height) (Table 2). T_bb_ was included in each of the top 8 candidate models and had the strongest effect on nest survival (Table 2) demonstrating that it was a more important variable than the vegetation structure variables that we examined as proxies for potential predation risk. However, significant improvements in AIC rankings were achieved when T_bb_ and vegetation variables were included together in candidate models (Table 2). Specifically, the top 2 candidate models received similar statistical support (DAIC <2) and included T_bb_ and angle of obstruction as well as T_bb_, angle of obstruction and vegetation height, respectively (Table 2). The four top candidate models accounted for 98% of the Akaike weight (w_i_) (Table 2) and were used to identify model-averaged coefficients used to solve for odds ratios and 95% confidence intervals. Odds ratios were 1.025 (95% CI, 1.00–1.04), 1.01 (95% CI, 1.00–1.02), 0.86 (95% CI, 0.54–1.38) and 1.0 (95% CI, 0.92–1.09) for T_bb_, angle of obstruction, visual obstruction and vegetation height, respectively. Additionally, differences in T_bb_ between nest fates were temporally explicit with the most pronounced variability occurring during mid-day and afternoon periods on extreme heat days (T_air_ ≥ 39°C) (Fig 4A). Interestingly, mean T_bb_ between the 48 successful and 27 unsuccessful nests on days with maximum T_air_ < 39°C (F_1, 805_ = 0.034, p = 0.85) (n = 75) (Fig 4B) were similar. However, mean T_bb_ at the 6 successful nests were on average 6 C°C cooler than at the 6 unsuccessful nests sampled on days experiencing maximum T_air_ ≥ 39°C (F _1,130_ = 6.56, p < 0.05) (n = 12) (Fig 4A).

**Table 2:** Logistic regression candidate model rankings for variables affecting northern bobwhite nest success at the Packsaddle WMA, Oklahoma, USA (2013–2014). Black bulb temperature (i.e., T_bb_), angle of obstruction, visual obstruction, and vegetation height (i.e., height) were variables included in candidate models.

Candidate Model	K^a^	AIC	DAIC	w_i_ ^b^
T_bb_ + Angle	3	1241.7	0.0	0.38
T_bb_ + Visual Obstruction + Height	4	1241.9	0.2	0.34
T_bb_ + Visual Obstruction + Angle	4	1243.7	2.2	0.13
Global	5	1243.7	2.2	0.13
T_bb_ + Visual Obstruction + Height	4	1249.4	7.5	< 0.01
T_bb_ + Visual Obstruction	3	1251.0	9.1	< 0.01
T_bb_	2	1251.6	9.7	< 0.01
T_bb_ + Height	3	1252.3	10.4	< 0.01
Angle Obstruction	2	1267.6	25.7	< 0.01
Angle Obstruction + Height	3	1268.8	27.1	< 0.01
Angle Obstruction + Visual Obstruction	3	1269.3	27.6	< 0.01
Visual Obstruction + Height	3	1271.4	29.7	< 0.01
Visual Obstruction	2	1271.4	29.7	< 0.01
Null	1	1272.4	30.7	<0.01
Height	2	1273.7	31.8	< 0.01

^a^Number of parameters

^b^Akaike weight

**Fig 5:**
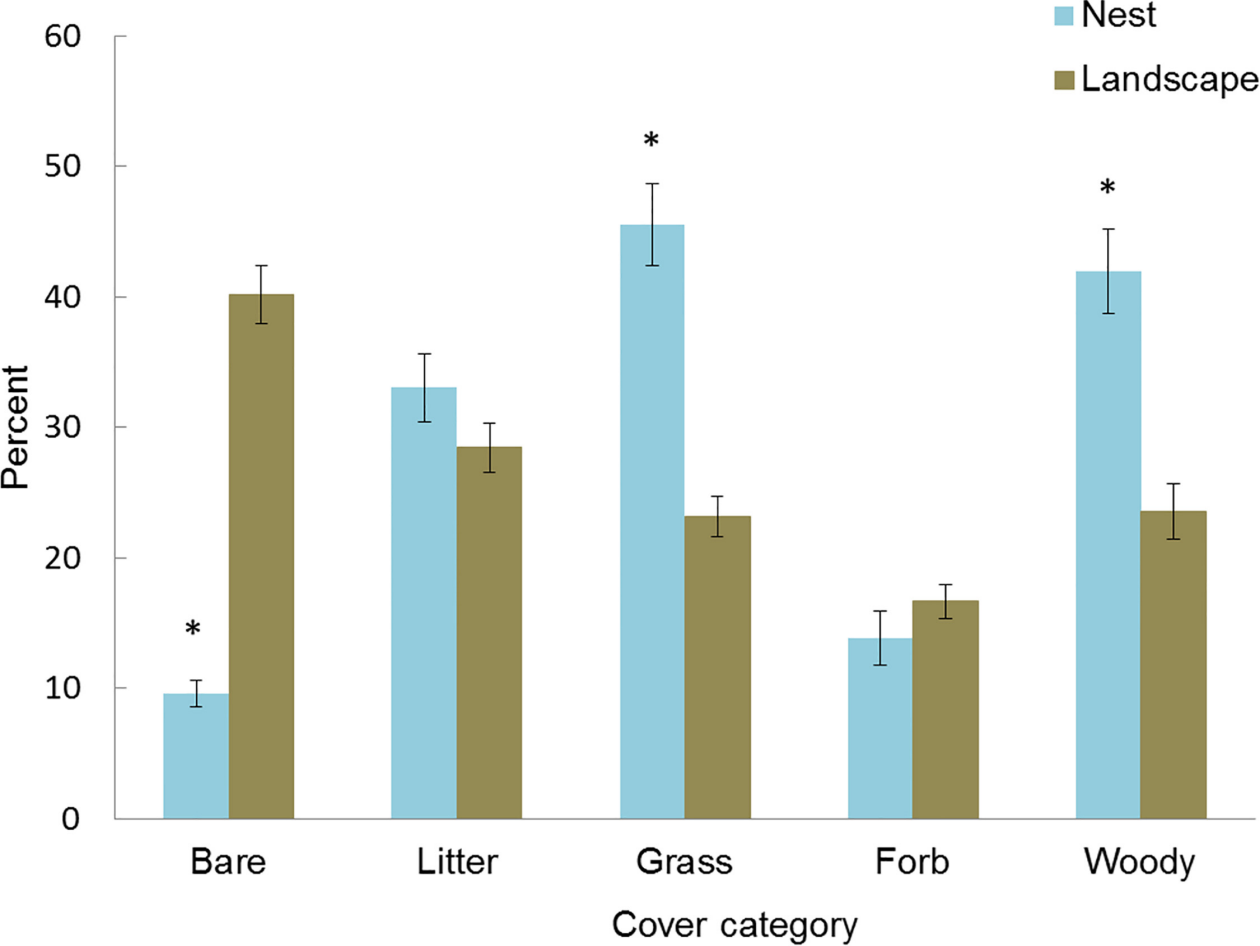
Vegetation characteristics vary among nest and landscape sites. Percent vegetation cover measured at northern bobwhite nest (n = 87) and landscape sites (n = 205) at the Packsaddle WMA, Oklahoma, USA (2013–2014). Asterisks denote significant differences at the p < 0.05 level within cover categories.

The moderated microclimates selected by bobwhites as nest sites also afforded different vegetation structure than those at landscape sites. For example, angle of obstruction (71.61° ± 2.4) was significantly greater at nest sites than at landscape sites (41.45° ± 1.9) (F_1, 290_ = 80.94, p < 0.0001). Similarly, lateral visual obstruction (6.81dm ± 0.20) at nest sites was also significantly greater than at landscape sites (5.96 dm ± 0.22) (F_1, 290_ = 5.23, p < 0.05). Moreover, greater percent grass and woody cover and less bare ground cover occurred at nest sites compared to landscape sites (Fig 5), however no differences were found between litter or forb cover. Mean vegetation height (±SE) was similar between successful (0.74 meters ± 0.03) and unsuccessful nests (0.73 meters ± 0.06) and each offered similar lateral visual concealment (F_1, 85_ = 1.72, p = 0.19) and overhead obstruction (F_1,85_ = 0.69, p = 0.41). When examining other fine scale vegetation parameters among successful and unsuccessful nests, we found no differences (p > 0.10) in any of the vegetation cover variables that were measured.

The study area consists of 50% herbaceous cover and 37% in low shrub cover (unpublished data) and of the 87 nests, 49.4% and 50.6% of the 87 nests were located in grass and shrub cover, respectively. Nest success was 58% in nests positioned in grass and 66% in nests positioned in shrubs. Nests located in shrub and grass cover provided similar T_bb_ on days with maximum T_air_ < 39°C (F_1, 805_ = 0.13, p = 0.72) (n = 75), however, shrub cover provided substantially cooler thermal conditions than grass cover on days when maximum T_air_ ≥ 39°C (F_1, 130_ = 9.26, p < 0.005) (n = 12).

**Fig 6:**
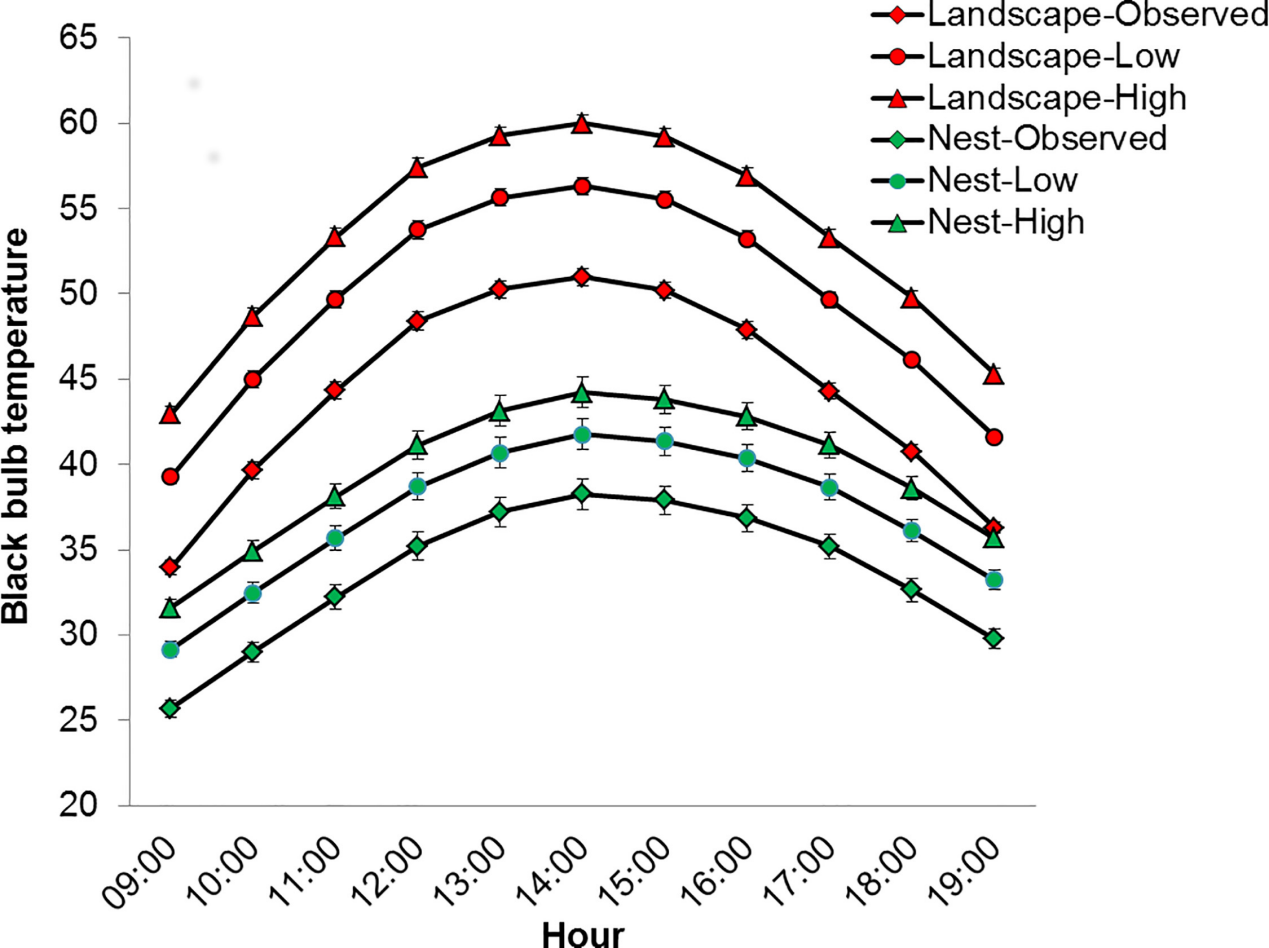
Increased duration and intensity of thermal constraints associated with future climate change. Black bulb temperature (T_bb_) (±SE) measured at northern bobwhite nest sites (green) and random landscape sites (red). Marker shape denotes observed conditions (square) and those associated with projected increases in T_air_ as predicted by the low (circle) and high (triangle) emission end of century scenario ensemble averages at the Packsaddle WMA, Oklahoma, USA, 2013–2014 (n = 7,008).

Simple linear models of T_bb_ as a function of T_air_ explained, 77% and 73% of the variation in site-specific T_bb_ measured at nest and landscape sites, respectively. Our models of T_bb_ associated with future climate change indicate that nesting bobwhites will face substantially greater T_bb_ for longer durations (Fig 6). Specifically, we found that future thermal conditions on the landscape could potentially exceed T_bb_ of 50°C from 12:00–16:00 for low emission scenarios and from 11:00–17:00 for high emission scenarios (Fig 6). However, while nest sites generally offered much less severe environments than those occurring on the landscape, nests sites will also potentially experience a substantial increase in exposure to thermal extremes for longer durations in the future. For example, under present conditions, mean T_bb_ at nest sites remained less than 39°C for the entire day but will exceed 39°C for at least 4 hours of the day under low emission scenarios and at least 6 hours of the day under high emission scenarios (Fig 6).

## Discussion

These results provide a linkage between thermal moderation as a component of landscape function and the biologically meaningful response of a ground-nesting bird species. We found that the thermal landscape was highly heterogeneous and provided an extensive variety of microclimates available to ground-nesting birds. Specifically, we observed that T_bb_ differences ranged up to 40°C among microhabitats across the landscape when T_air_ > 35°C, demonstrating that certain portions of the landscape moderate thermal environments considerably more than others. Our findings also revealed the potential for high thermal extremes (> 70°C) occurring on the landscape, further substantiating the importance of thermal choices for ground-dwelling birds to obtain suitable microhabitats for nesting. Moreover, a reduction in thermal heterogeneity or loss of thermally-buffered microhabitats would likely have negative impacts on ground-nesting bird species once a threshold of intolerable thermal space was reached. Therefore, management practices that maintain the heterogeneity of vegetation and thermal environments inherent in shrub landscapes will likely benefit nesting bobwhites by promoting thermally complex matrices of shrubs and grasses, some of which serve as locations for nest sites. Importantly, our models show that reductions of thermally suitable space and greater heat extremes will increasingly confront nesting bobwhites as a result of climate change, suggesting that the impacts of thermal environments on critical reproductive stages is a notable concern for future conservation of species constrained to near ground climates.

The landscape sampled in this study was prone to extreme T_bb_ that regularly exceeded 50°C during several hours of the day. These findings have major biological implications given that the lethal body temperature for most birds, including bobwhites is 47°C [8, 57] and that the lethal egg temperature for bobwhites (50% mortality) has been reported for eggs exposed to 46°C for as little as 1 hour [58]. In response to these thermal extremes, bobwhite nest sites offered cooler and less variable thermal conditions than those on the surrounding landscape. Furthermore, ground level T_bb_ at paired control sites was subject to substantial temporal variation and displayed the potential for extremes; however, proximate extremes were greatly mitigated at nest sites. Differences between nest and landscape site T_bb_ were especially greatest on the hottest days (by on average more than 6°C), demonstrating that thermally-buffered sites likely aided in reducing thermal stress during the reproductive cycle. Using a similar approach, a recent study in the tall grass prairie of Oklahoma observed that greater prairie-chickens (*Tympanuchus cupido*) exhibited similar responses to thermal environments as bobwhites in this study did; specifically, sites selected for nests substantially moderated landscape thermal conditions and successful nests exhibited cooler T_bb_ than unsuccessful nests [32]. These similarities demonstrate that nest site selection by ground-nesting birds in two different vegetation communities has an explicit thermal context and reinforces the importance of fine scale thermal heterogeneity for the future persistence of these species.

Habitat selection has been shown to result from the synergistic effects environmental patterns as well as vegetation structure which confer impacts on animals that are scale dependent [85–87]. Although these factors can often be confounded [70], examining microclimatic conditions at selected locations and the surrounding landscape is a step towards understanding the spatio-temporal trade-offs associated with habitat selection [32]. Nest sites in our study were less exposed overhead, provided significantly greater grass and woody cover than those predominately available on the landscape, and were correlated with moderated microclimates. Our finding that T_bb_ was the better single predictor of nest fate suggests that nest success during extreme heat may have been a result of moderated microclimate rather than protection from nest predators. However, top models included both T_bb,_ angle of obstruction and vegetation height, demonstrating the complexity of the microclimate-predation risk tradeoffs involved in nesting.

Long term temperature averages can be informative for broadly assessing bird population responses to climate [88], however, the regularity and intensity of thermal extremes occurring at hourly or diurnal scales are also critical [89], yet less studied. Interestingly, we found that nest site T_bb_ was similar between successful and unsuccessful nests on less extreme days (<39°C), however, successful nests moderated T_bb_ by more than 6°C compared to unsuccessful nests on days of extreme heat (≥ 39°C). Although our methodology precluded us from gaining a direct linkage between nest fate and T_bb_ at the time of nest failures, the contrast of the pattern that we observed based on relative T_bb_ differences was stark and suggests that further research is needed. The potential importance of this pattern is further elevated given that the range of thermal conditions ensuring successful incubation is narrow and short bouts of extreme high temperatures can be lethal to embryos [57, 58]. In addition, differences in site-specific microclimate conditions among successful and unsuccessful nests during extreme heat may be highly impactful they are likely missed by studies assessing environmental conditions at coarser scales.

A key component to understanding the ecological impacts of future climate change will require knowledge on how critical life history periods may be influenced by proximate thermal environments [20, 90]. Broad scale climate change will likely shift the juxtaposition of fine scale thermal regimes relevant to organisms [2, 91], yet the magnitude and spatial distribution of these shifts are poorly understood [28]. We found that although broad scale climate greatly influences microclimates, biologically relevant thermal buffering decoupled nest sites from regional conditions (i.e., meteorological station data) as well as microclimates on the surrounding landscape. However, under current conditions nests showed the potential for reaching extremely high mean T_bb_ on occasion and our models indicate that thermal extremes will become increasingly exacerbated with the higher projected temperatures. For example, we found that under high emission scenarios mean T_bb_ experienced at nest sites may reach 44°C at nest sites and 60°C at landscape sites. Given that bobwhites have been shown to avoid thermal space ≥ 39°C, nest sites will commonly experience suboptimal T_bb_. Therefore, incubating bobwhites and their embryos will be exposed to substantially more extreme T_bb_ for longer durations despite the relative thermal buffering provided by nest sites. Furthermore, given that temperatures ≥ 50°C exceed both the lethal body temperature for birds [8] and the temperature threshold that precludes most biotic life [72, 92], our models suggest that few microhabitats across the landscape will be available to ground nesting avifauna during summer.

Answering ecological questions has historically been hindered by examinations of broad scale patterns that lack local scale relevance [13, 93]. Moreover, the common practice of assessing scales larger than those experienced by study organisms can inhibit our knowledge about how individuals exploit thermal landscapes, especially in the face of climate change [23]. We provide evidence that heterogeneous landscapes provide microhabitats that moderate thermal extremes and were consequently selected as nest sites by bobwhites. By incorporating detailed assessments of thermal environments as a part of the experimental design, future studies would be better suited to bridge the gap between broad scale climate patterns and the microclimates that organisms experience. For example, thermally induced ecological traps could reduce populations through the inhibition of required for reproductive success [94], especially in cases where preferred microclimates become fewer and farther between. Therefore, identifying whether animals will respond to increased future thermal stresses by dispersing to tolerable environments, adapting to thermally-stressful conditions [95], or perishing will be critical to guide conservation efforts. Our findings show that diurnal and hourly thermal constraints and heat load exposure on ground-nesting birds will likely be exacerbated due to increased high heat associated with climate change. As a result, the need for an increased conservation focus on the mitigation of thermal extremes is likely urgent. Furthermore, these findings demonstrate that the management of thermal space for ground-nesting birds should focus on providing structural complexity that allows species to make hierarchical nest selection decisions at both landscape and fine scales.

## References

[1] Smith RL, Smith TM. Elements of Ecology. Fourth Edition Benjamin/Cummings Science Publications; 2000.

[2] Angilletta MJ. Thermal adaptation: a theoretical and empirical synthesis Oxford University Press; 2009.

[3] Dawson RD, Lawrie CC, O’Brien EL. The importance of microclimate variation in determining size, growth and survival of avian offspring: experimental evidence from a cavity nesting passerine. Oecologia. 2005; 144: 499–507. 15891832 10.1007/s00442-005-0075-7

[4] Weber SB, Broderick AC, Groothuis TG, Ellick J, Godley BJ, Blount JD. Fine-scale thermal adaptation in a green turtle nesting population. Proc R Soc Lond B Biol Sci. 2011; 279: 1077–1084. 10.1098/rspb.2011.1238 PMC3267129 21937495

[5] Peterman WE, Semlitsch RD. Fine-scale habitat associations of a terrestrial salamander: the role of environmental gradients and implications for population dynamics. PloS One. 2013; 8: e62184 10.1371/journal.pone.0062184 23671586 PMC3646024

[6] Lawson C R, Bennie J, Hodgson JA, Thomas CD, Wilson RJ. Topographic microclimates drive microhabitat associations at the range margin of a butterfly. Ecography. 2014; 37: 732–740.

[7] Zhao F., Zhang W, Hoffmann AA, Ma CS. 2014 Night warming on hot days produces novel impacts on development, survival and reproduction in a small arthropod. J Anim Ecol. 2014; 83: 769–778. 10.1111/1365-2656.12196 24372332

[8] Kendeigh SC. Energy responses of birds to their thermal environments. The Wilson Bulletin. 1969; 81: 441–449.

[9] Salzman AG. The selective importance of heat stress in gull nest location. Ecology. 1982; 63: 742–751.

[10] Webb DR. Thermal tolerance of avian embryos: a review. Condor. 1987; 89: 874–898.

[11] Duarte H, Tejedo M., Katzenberger M, Marangoni F, Baldo D, Beltrán JF, et al Can amphibians take the heat? Vulnerability to climate warming in subtropical and temperate larval amphibian communities. Glob Chang Biol. 2012; 18: 412–421.

[12] Pike DA. Forecasting the viability of sea turtle eggs in a warming world. Glob Chang Biol. 2014; 20: 7–15. 10.1111/gcb.12397 24106042

[13] Kotliar NB, Wiens JA. Multiple scales of patchiness and patch structure: a hierarchical framework for the study of heterogeneity. Oikos. 1990; 59: 253–260.

[14] McGill BJ. Matters of scale. Science. 2010; 328,: 575–576. 10.1126/science.1188528 20431001

[15] van Beest FM, Van Moorter B, Milner JM. Temperature-mediated habitat use and selection by a heat-sensitive northern ungulate. Anim Behav. 2012; 84: 723–735.

[16] Dubois Y, Blouin‐Demers G, Shipley B, Thomas D. Thermoregulation and habitat selection in wood turtles Glyptemys insculpta: chasing the sun slowly. J Anim Ecol. 2009; 78: 1023–1032. 10.1111/j.1365-2656.2009.01555.x 19426255

[17] Schofield G, Bishop CM, Katselidis KA, Dimopoulos P, Pantis JD, Hays GC. Microhabitat selection by sea turtles in a dynamic thermal marine environment. J Anim Ecol. 2009; 78: 14–21. 10.1111/j.1365-2656.2008.01454.x 18699794

[18] Cunningham SJ, Kruger AC, Nxumalo MP, Hockey PA. Identifying biologically meaningful hot-weather events using threshold temperatures that affect life-history. PloS One. 2013; 8: e28492. 10.1371/journal.pone.0082492 PMC3861557 24349296

[19] Austin MP, Van Niel KP. Improving species distribution models for climate change studies: variable selection and scale. J Biogeogr. 2011; 38: 1–8.

[20] Potter KA, Woods HA, Pincebourde S. Microclimatic challenges in global change biology. Glob Chang Biol. 2013; 19: 2932–2939. 10.1111/gcb.12257 23681970

[21] Hannah L, Flint L, Syphard AD, Moritz MA, Buckley LB, McCullough IM. Fine-grain modeling of species’ response to climate change: holdouts, stepping-stones, and microrefugia. Trends Ecol Evol. 2014; 29: 390–397. 10.1016/j.tree.2014.04.006 24875589

[22] Logan ML, Huynh RK, Precious RA, Calsbeek R.G. The impact of climate change measured at relevant spatial scales: new hope for tropical lizards. Glob Chang Biol. 2013; 19: 3093–3102. 10.1111/gcb.12253 23661358

[23] Varner J, Dearing M.D. The importance of biologically relevant microclimates in habitat suitability assessments. PloS One. 2014; 9: e104648 10.1371/journal.pone.0104648 25115894 PMC4130583

[24] Christensen NL. 1997. Managing for heterogeneity and complexity on dynamic landscapes In: Pickett STA, Ostfeld RS, Shachak M, Likens GE, editors. The ecological basis of conservation: heterogeneity, ecosystems, and biodiversity. Chapman and Hall; 1997 pp. 167–186.

[25] Wiens JA. 1997. The emerging role of patchiness in conservation biology In: Pickett STA, Ostfeld RS, Shachak M, Likens GE, editors. The ecological basis of conservation: Heterogeneity, Ecosystems, and Biodiversity. Chapman and Hall; 1997 pp. 93–107.

[26] Limb RF, Fuhlendorf SD, Townsend DE. Heterogeneity of thermal extremes: driven by disturbance or inherent in the landscape. Environ Manage. 2009; 43: 100–106. 10.1007/s00267-008-9147-x 18491182

[27] Rosenberg NJ, Blad BL, Verma SB. Microclimate: the biological environment John Wiley and Sons; 1983.

[28] Sears MW, Raskin E, Angilletta MJ. The world is not flat: defining relevant thermal landscapes in the context of climate change. Integr Comp Biol. 2011; 51: 666–675. 10.1093/icb/icr111 21937668

[29] Perfecto I, Vandermeer J. Microclimatic changes and the indirect loss of ant diversity in a tropical agroecosystem. Oecologia. 2012; 108: 577–582. 10.1007/BF00333736 28307876

[30] Patten MA, Smith-Patten BD. Testing the microclimate hypothesis: Light environment and population trends of Neotropical birds. Biol Conserv. 2012; 155: 85–93.

[31] Walsberg GE. Nest-site selection and the radiative environment of the Warbling Vireo. Condor. 1981; 83: 86–88.

[32] Hovick TJ., Elmore RD, Allred BW, Fuhlendorf SD, Dahlgren DK. Landscapes as a moderator of thermal extremes: a case study from an imperiled grouse. Ecosphere. 2014; 5: 1–12.

[33] Hovick TJ, Elmore RD, Fuhlendorf SD, Dahlgren D.K. Weather Constrains the Influence of Fire and Grazing on Nesting Greater Prairie-Chickens. Rangeland Ecology & Management. 2015; 68: 186–193.

[34] Cowles RB, Bogert CM. A preliminary study of the thermal requirements of desert reptiles. Bulletin of American Museum of Natural History. 1944; 83: 261–296.

[35] Shine R. Life-history evolution in reptiles. Annual Rev Ecol Evol Syst. 2005; 3: 23–46.

[36] Ford KR, Ettinger AK, Lundquist JD, Raleigh MS, Lambers JHR. Spatial Heterogeneity in Ecologically Important Climate Variables at Coarse and Fine Scales in a High-Snow Mountain Landscape. PloS One. 2013; 8: e65008 10.1371/journal.pone.0065008 23762277 PMC3676384

[37] Tieleman IB, Noordwijk HJ, Williams JB. 2008. Nest site selection in a hot desert: trade-off between microclimate and predation risk? Condor. 2008; 110: 116–124.

[38] Angilletta MJ, Sears MW, Pringle RM. Spatial dynamics of nesting behavior: lizards shift microhabitats to construct nests with beneficial thermal properties. Ecology. 2009; 90: 2933–2939. 19886501 10.1890/08-2224.1

[39] Huang WS, Pike DA. Climate change impacts on fitness depend on nesting habitat in lizards. Funct Ecol. 2011; 25: 1125–1136.

[40] DuRant SE, Hopkins WA, Hepp GR, Walters JR. Ecological, evolutionary, and conservation implications of incubation temperature‐dependent phenotypes in birds. Biol Rev Camb Philos Soc. 2013; 88: 499–509. 10.1111/brv.12015 23368773

[41] Hansell MH, and Deeming C. Location, structure and function of incubation sites In: Deeming DC, editors. Avian incubation: behaviour, environment and evolution. Oxford University Press; 2002 pp. 8–27.

[42] Mainwaring MC, Deeming DC, Jones CI, Hartley I R. 2014. Adaptive latitudinal variation in Common Blackbird Turdus merula nest characteristics. Ecol Evol. 2002; 4: 851–861. 10.1002/ece3.952 PMC3967909 24683466

[43] Ricklefs RE, Hainsworth FR. Temperature dependent behavior of the cactus wren. Ecology. 1968; 49: 227–233.

[44] Huey RB. 1991. Physiological consequences of habitat selection. Am Nat. 1991; 137: S91–S115.

[45] Telemeco RS, Elphick MJ, Shine R. Nesting lizards (*Bassiana duperreyi*) compensate partly, but not completely, for climate change. Ecology. 2009; 90: 17–22. 19294908 10.1890/08-1452.1

[46] Bennett AF, Dawson WR, Putnam RW. Thermal environment and tolerance of embryonic Western Gulls. Physiol Zool. 1981; 54: 146–154.

[47] Gloutney ML and Clark RG. Nest-site selection by Mallards and Blue-winged Teal in relation to microclimate. Auk. 1997; 114: 381–395.

[48] Wiebe KL and Martin K. Costs and benefits of nest cover for ptarmigan: changes within and between years. Anim. Behav. 1998; 56: 1137–1144 9819329 10.1006/anbe.1998.0862

[49] Martin TE. Abiotic vs. biotic influences on habitat selection of coexisting species: climate change impacts? Ecology. 2001; 82:175–188.

[50] Goldstein DL. 1984. The thermal environment and its constraint on activity of desert quail in summer. Auk. 1984; 101: 542–550.

[51] Guthery FS, Land CL, Hall BW. Heat loads on reproducing bobwhites in the semiarid subtropics. J Wildl Manage. 2001; 65: 111–117.

[52] Patten MA, Wolfe DH, Shochat E, Sherrod SK. 2005. Effects of microhabitat and microclimate selection on adult survivorship of the lesser prairie-chicken. J Wildl Manage. 2005; 69: 1270–1278.

[53] Albright TP, Pidgeon AM, Rittenhouse CD, Clayton MK, Wardlow BD, Flather CH, et al Combined effects of heat waves and droughts on avian communities across the conterminous United States. Ecosphere. 2010; 1: 1–22.

[54] Johnson DB, Guthery FS. Loafing coverts used by northern bobwhites in subtropical environments. J Wildl Manage. 1988; 52: 464–469.

[55] Guthery FS, Koerth NE, Smith DS. Reproduction of northern bobwhites in semiarid environments. J Wildl Manage. 1988; 52: 144–149.

[56] Guthery FS, Rybak AR, Fuhlendorf SD, Hiller TL, Smith SG, Puckett WH, et al Aspects of the thermal aspects of bobwhites in north Texas. Wildlife Monographs. 2005; 159: 1–36.

[57] Guthery FS. On Bobwhites. Texas A&M University Press; 2000.

[58] Reyna KS, Burggren WW. Upper lethal temperatures of Northern Bobwhite embryos and the thermal properties of their eggs. Poult Sci. 2012; 91: 41–46. 10.3382/ps.2011-01676 22184426

[59] Sauer JR, Hines JE, Fallon JE, Pardeick KL, Ziolkowakis DJ Jr., Link WA. The North American Breeding Bird Survey Results and Analysis 1966–2009. Version 3.23.2011 USGS Patuxent Wildlife Research Center; 2011.

[60] Woods HA, Dillon ME, Pincebourde S. The roles of microclimatic diversity and of behavior in mediating the responses of ectotherms to climate change. J Therm Biol. 2014; 1–10. 10.1016/j.jtherbio.2014.10.002 26615730

[61] Peterson R, Boyd CS. Ecology and management of sand shinnery oak communities: a literature review. United States Forest Service General Technical Report RMRS-GTR-16; 1998.

[62] DeMaso SJ, Peoples AD, Cox SA, Parry ES. Survival of northern bobwhite chicks in western Oklahoma. J Wildl Manage. 1997; 61: 846:853.

[63] Vermeire LT, Wester DB. Shinnery oak poisoning of rangeland cattle: causes, effects & solutions. Rangelands. 2001; 23: 19–21.

[64] Wiedeman VE, Penfound WT. A preliminary study of the shinnery in Oklahoma. Southwest Nat. 1960; 5: 117–122.

[65] Oklahoma Mesonet. Arnett station Rainfall 1994–2013; Available: http://www.mesonet.org/index.php/weather/monthly_rainfall_table/ARNE/estimated

[66] Arndt D. The Climate of Oklahoma. OCS Climate Information Group; 2003 Available: http://cig.mesonet.org/climateatlas/doc60.html.

[67] Oklahoma Mesonet. Arnett station temps 1994–2013; Available: http://www.mesonet.org/index.php/weather/mesonet_averages_maps#y=2012&m=7&p=tair_mx&d=false.

[68] Campbell GS, Norman JM. An introduction to environmental biophysics Springer; 1998.

[69] Allred BW, Fuhlendorf SD, Hovick TJ, Elmore RD, Engle DM, Joern A. Conservation implications of native and introduced ungulates in a changing climate. Glob Chang Biol. 2013; 19, 1875–1883. 10.1111/gcb.12183 23505266

[70] Hiller TL, Guthery FS. Microclimate versus predation risk in roost and covert selection by bobwhites. J Wildl Manage. 2005; 69: 140–149.

[71] Dzialowski EM. Use of operative temperature and standard operative temperature models in thermal biology. J Therm Biol. 2005; 30: 317–334.

[72] Calder WA, King JR. Thermal and caloric relations of birds. Avian Biology. 1974; 4: 259–413.

[73] With KA, Webb DR. Microclimate of ground nests: the relative importance of radiative cover and wind breaks for three grassland species. Condor. 1993; 95: 401–413.

[74] Robertson BA. Nest-site selection in a postfire landscape: do parents make tradeoffs between microclimate and predation risk?. Auk. 2009; 126: 500–510.

[75] Martin TE. Are microhabitat preferences of coexisting species under selection and adaptive? Ecology. 1998; 79: 656–670.

[76] Daubenmire R. A canopy coverage method of vegetational analysis. Northwest Science. 1959; 33: 43–64.

[77] Nudds TD. Quantifying the vegetative structure of wildlife cover. Wildl Soc Bull. 1977; 5: 113–117.

[78] Guthery FS, Doerr TB, Taylor MA. Use of a profile board in sand shinnery oak communities. Journal of Range Management. 1981; Archives 34: 157–158.

[79] Kopp SD, Guthery FS, Forrester ND, Cohen WE. Habitat selection modeling for northern bobwhites on subtropical rangeland. J Wildl Manage. 1998; 62, 884–895.

[80] Saalfield ST, Conway WC, Haukos DA, Johnson WP. Snowy Plover nest site selection, spatial patterning, and temperatures in the Southern High Plains of Texas. J Wildl Manage. 2012; 76: 1703–1711.

[81] Burnham KP, and Anderson DR. Model selection and multimodel inference: a practical information-theoretic approach Springer Science & Business Media; 2002.

[82] Zar JH. Biostatistical Analysis. 2nd ed. Prentice Hall; 1984.

[83] Forrester ND, Guthery FS, Kopp SD, Cohen WE. Operative temperature reduces habitat space for northern bobwhites. J Wildl Manage. 1998; 62: 1506–1511.

[84] Girvetz EH, Zganjar C, Raber GT, Maurer EP, Kareiva P. Applied Climate-Change Analysis: The Climate Wizard Tool. PLoS One. 2009; 4: e8320 10.1371/journal.pone.0008320 20016827 PMC2790086

[85] Avgar T, Mosser A, Brown GS, Fryxell JM. Environmental and individual drivers of animal movement patterns across a wide geographical gradient. J Anim Ecol. 2013; 82: 96–106. 10.1111/j.1365-2656.2012.02035.x 23020517

[86] Van Moorter B, Bunnefeld N, Panzacchi M, Rolandsen CM, Solberg EJ, Sæther B-E. Understanding scales of movement: animals ride waves and ripples of environmental change. J Anim Ecol. 2013; 82: 770–780. 10.1111/1365-2656.12045 23414218

[87] Seabrook L, McAlpine C, Rhodes J, Baxter G, Bradley A, Lunney D. Determining range edges: habitat quality, climate or climate extremes? Divers Distrib. 2014; 20: 95–106.

[88] La Sorte FA, Jetz W. Avian distributions under climate change: towards improved projections. J Exp Biol. 2010; 213: 862–869. 10.1242/jeb.038356 20190111

[89] McKechnie AE, Hockey PR, Wolf B.O. Feeling the heat: Australian landbirds and climate change. Emu. 2012; 112: 1–7.

[90] Cavallo C, Dempster T, Kearney MR, Kelly E, Booth D, Hadden KM, Jessop TS. Predicting climate warming effects on green turtle hatchling viability and dispersal performance. Funct Ecol. 2015; 1–11.

[91] Davies ZG, Wilson RJ, Coles S, Thomas CD. Changing habitat associations of a thermally constrained species, the silver‐spotted skipper butterfly, in response to climate warming. J Anim Ecol. 2006; 75: 247–256. 16903062 10.1111/j.1365-2656.2006.01044.x

[92] Larcher W. Physiological plant ecology Second Edition Springer-Verlag; 1991.

[93] Forman RTT, Godron M. Landscape Ecology. John Wiley & Sons; 1986.

[94] Wong BB, Candolin U. Behavioral responses to changing environments. Behav Ecol. 2015; 26: 665–673.

[95] Moritz C, Agudo R. 2013. The future of species under climate change: resilience or decline? Science. 2013; 341: 504–508. 10.1126/science.1237190 23908228

